# Supplementary material for: The Influence of Ligands on the Pd-Catalyzed Diarylation of Vinyl Esters
Source: Molecules. 2024 May 11;29(10):2268. doi: 10.3390/molecules29102268 (PMC11123883; doi:10.3390/molecules29102268)

Supporting Information for:

The influence of ligands on the Pd-catalyzed diarylation of vinyl esters.

Anna Brodzka [1,*], Dominik Koszelewski [1], Anna Trzeciak [2], Lena Ruzik [3], Malgorzta Grela [1] and Ryszard Ostaszewski [1,*]

1 Institute of Organic Chemistry, Polish Academy of Sciences, Kasprzaka 44/52, 01-224 Warsaw, Poland;
2 Faculty of Chemistry, University of Wrocław, F. Joliot-Curie 14, 50-383 Wrocław, Poland
3 Faculty of Chemistry, Warsaw University of Technology, Noakowskiego 3, 00-664, Warsaw, Poland
* Correspondence: anna.brodzka@icho.edu.pl (A.B.), ryszard.ostaszewski@icho.edu.pl

1,2-diphenylethyl acetate (3)

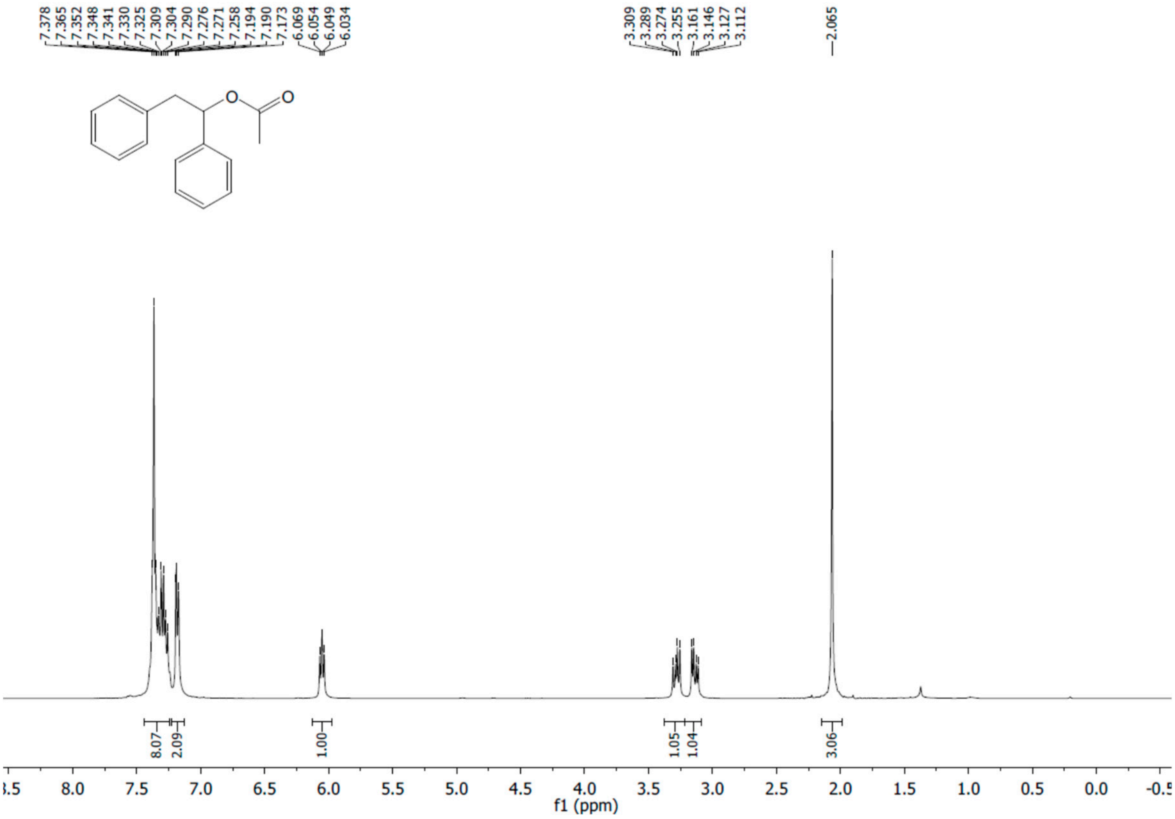

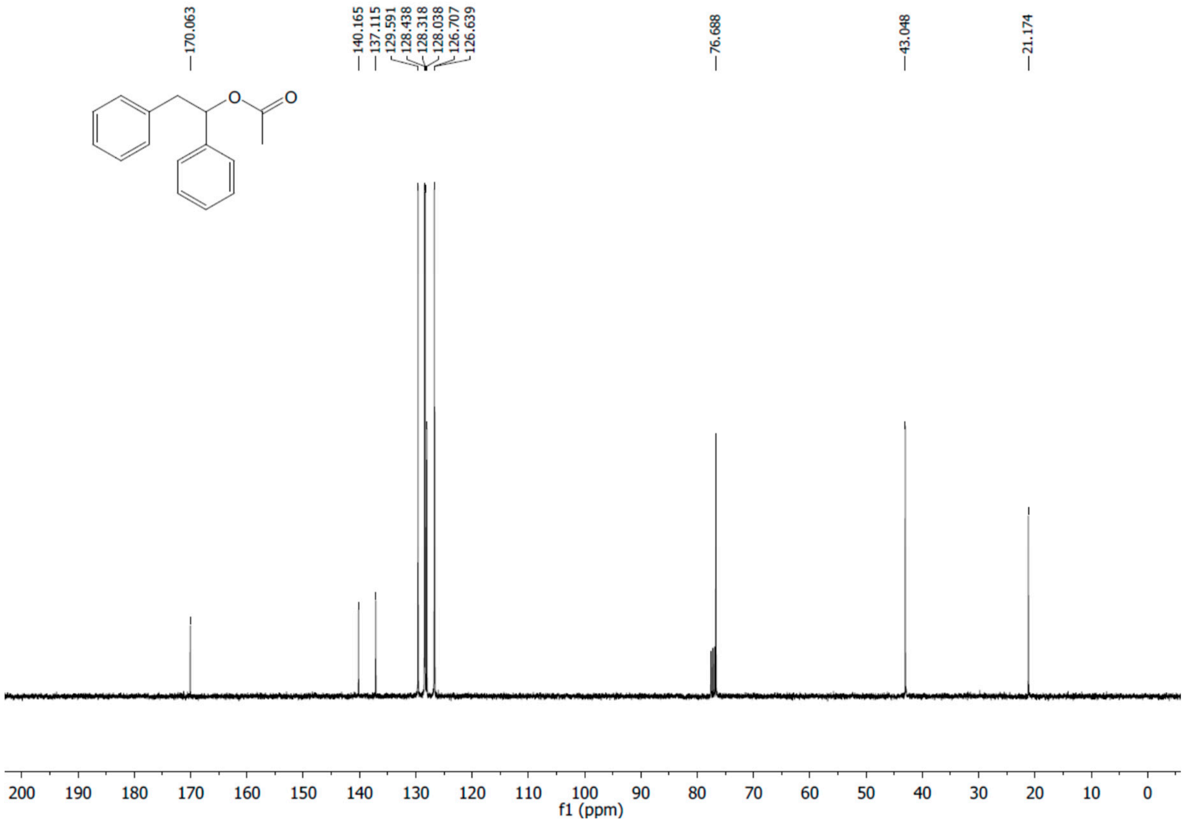

Stilbene (4)

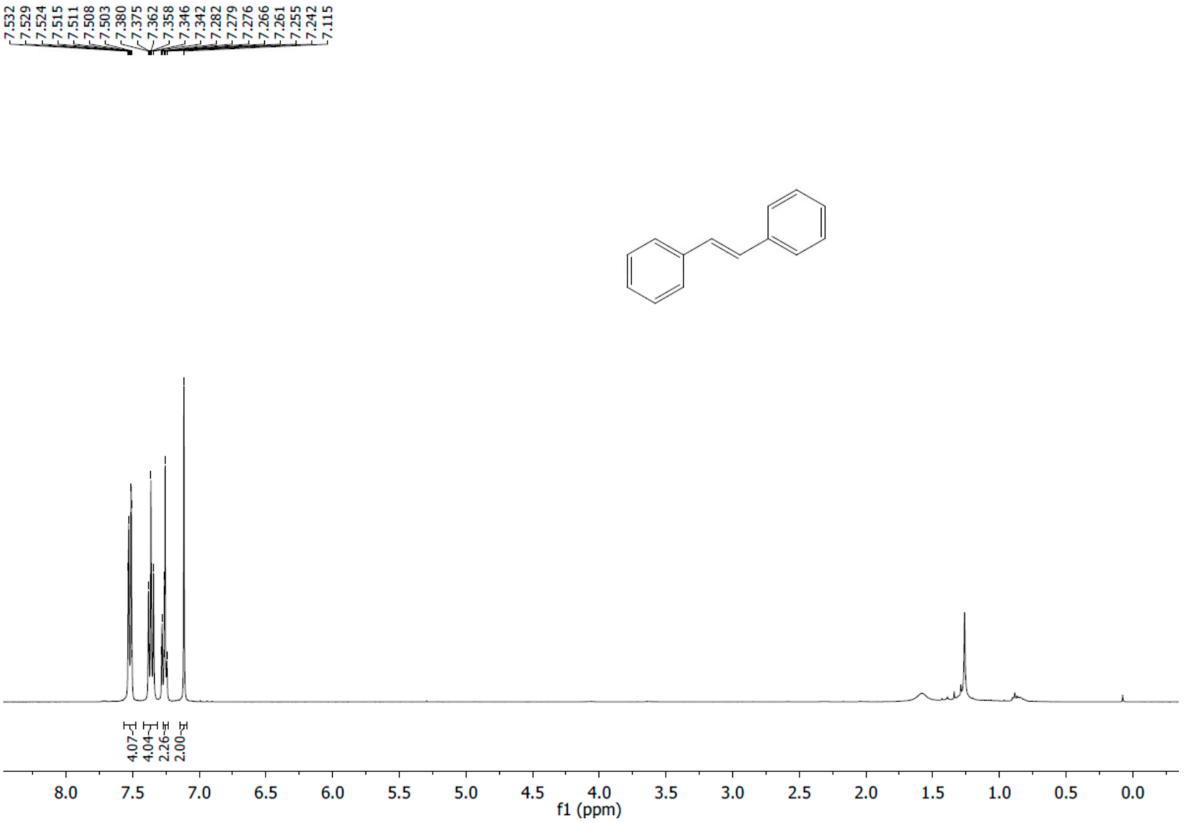
7.532
7.529
7.524
7.515
7.511
7.508
7.503
7.380
7.375
7.362
7.358
7.346
7.342
7.282
7.279
7.276
7.266
7.261
7.255
7.242
7.115
4.07
4.04
2.26
2.00
f1 (ppm)

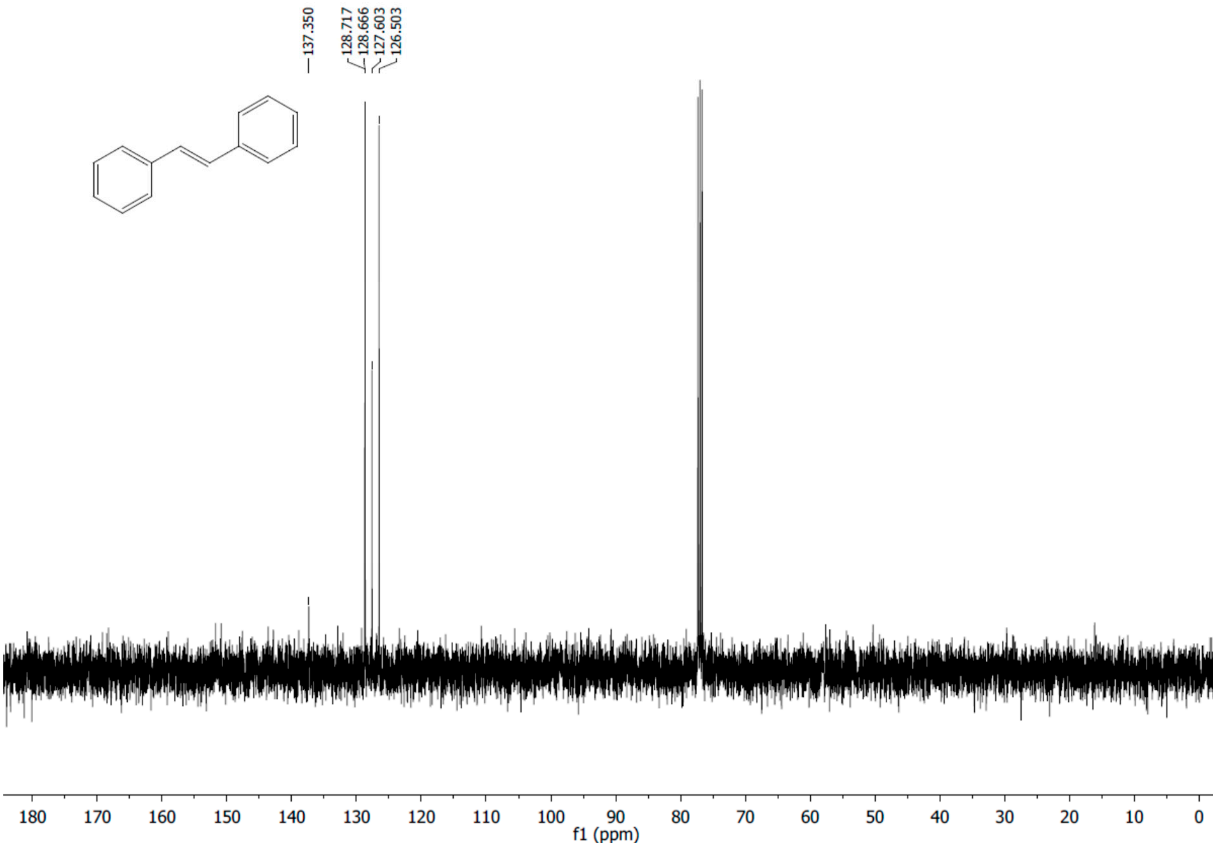
137.350
128.717
128.666
127.603
126.503
f1 (ppm)

2-phenyl-1,4-benzoquinone (5)

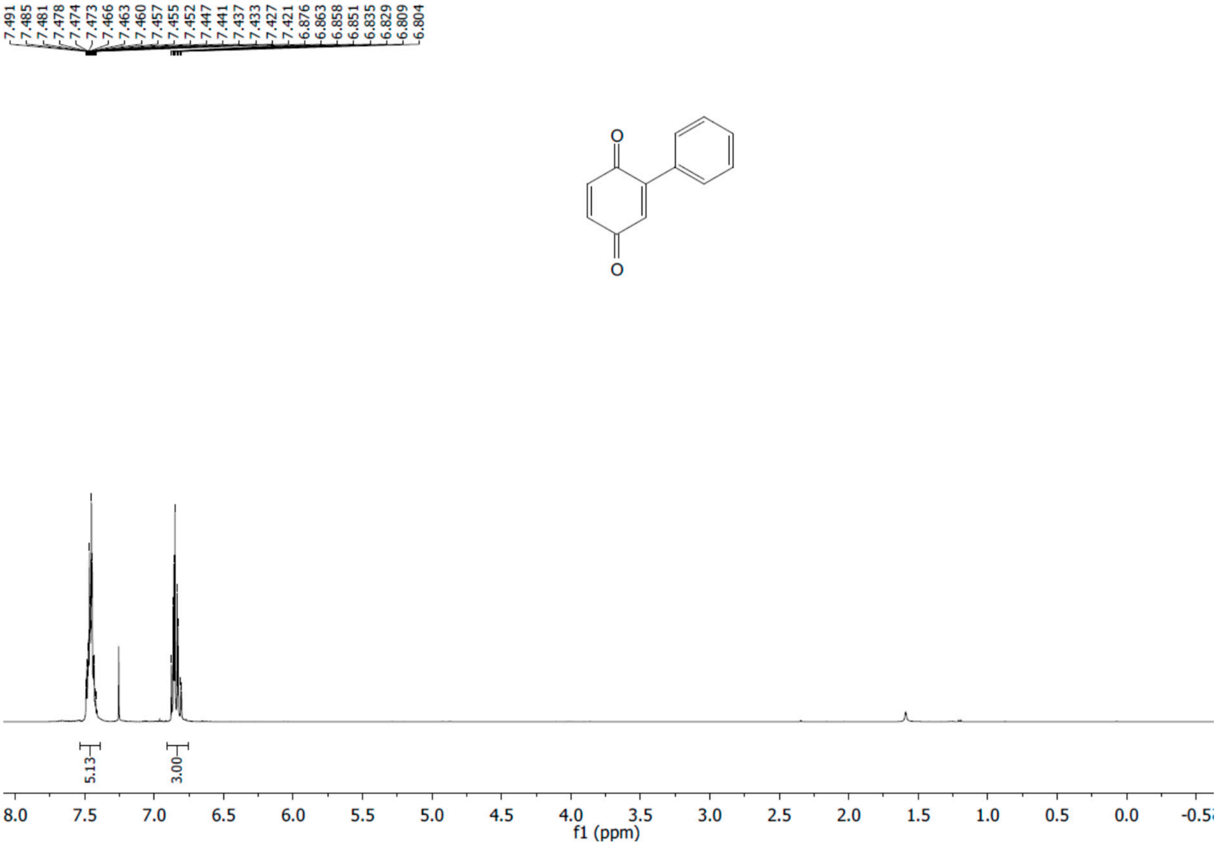

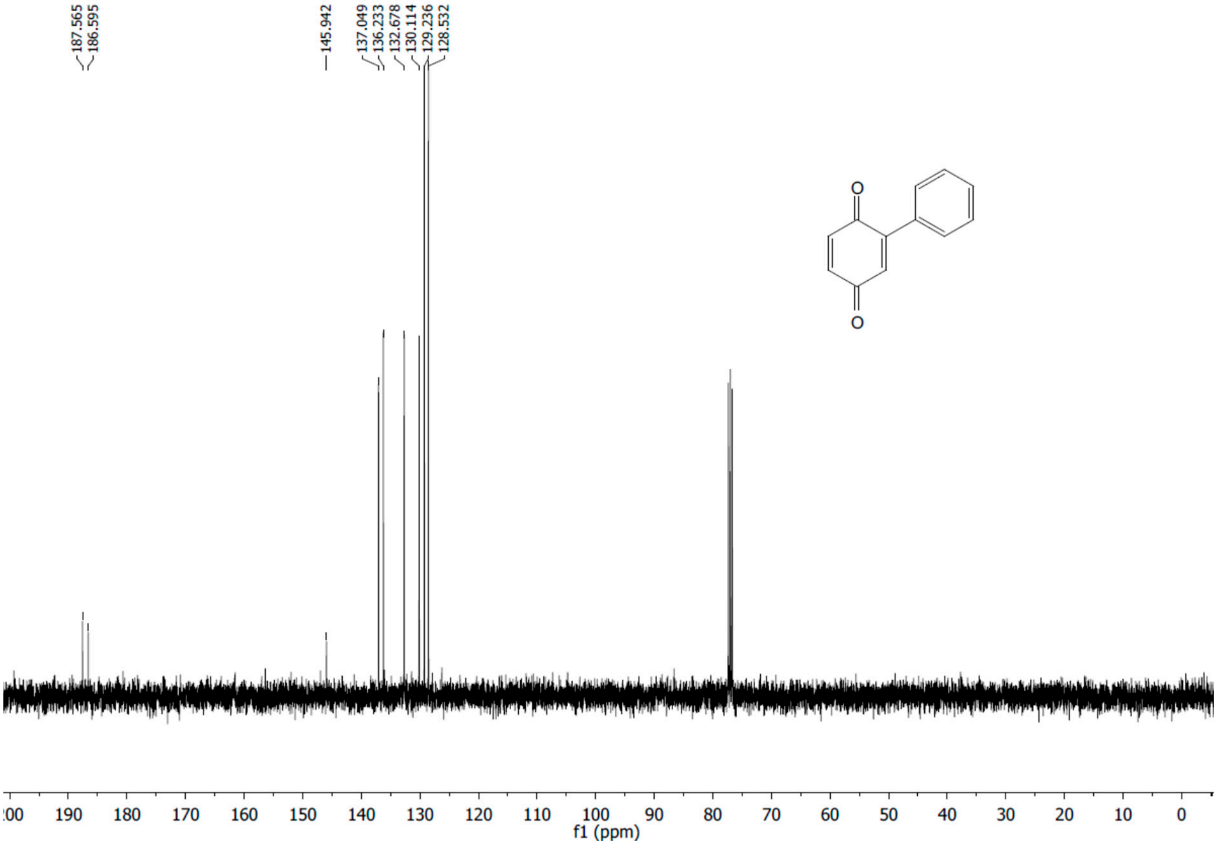

1,2-bis(3,4-dimethoxyphenyl)ethyl acetate (6)

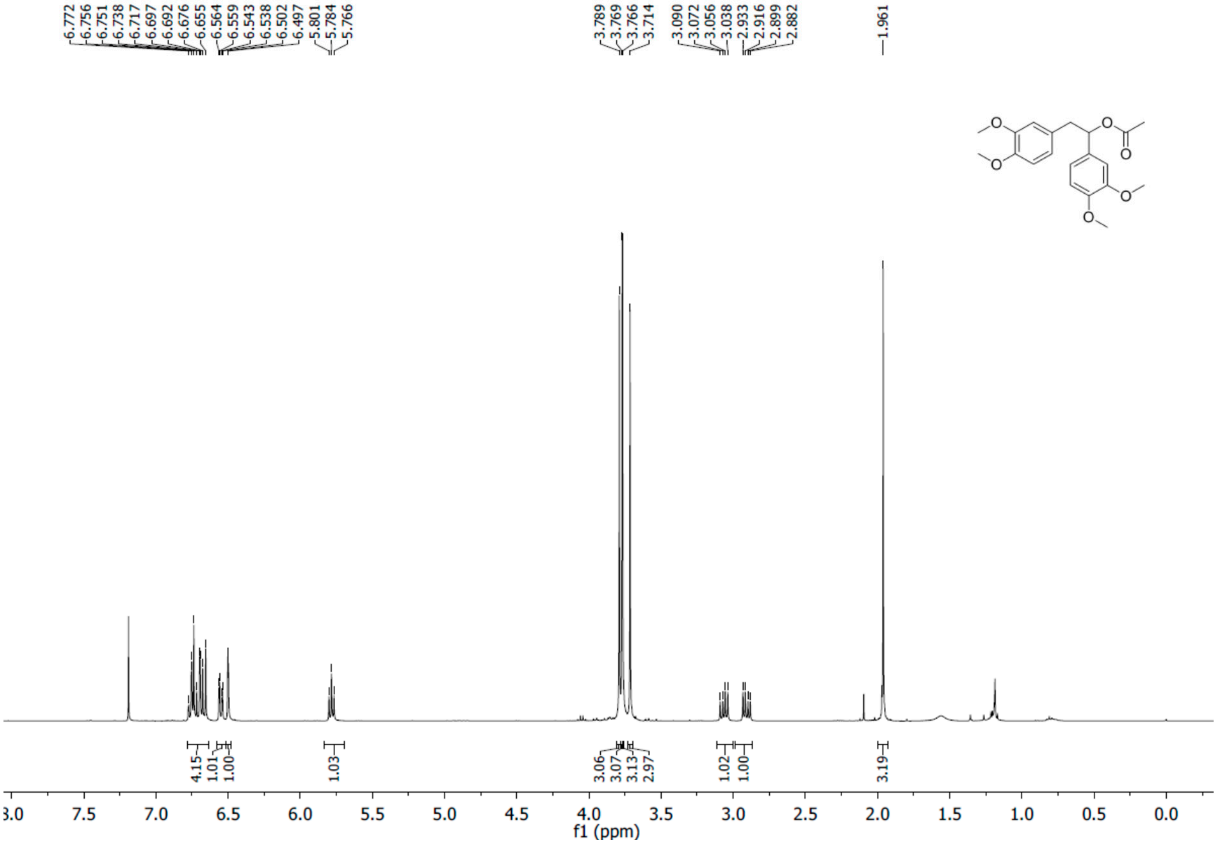

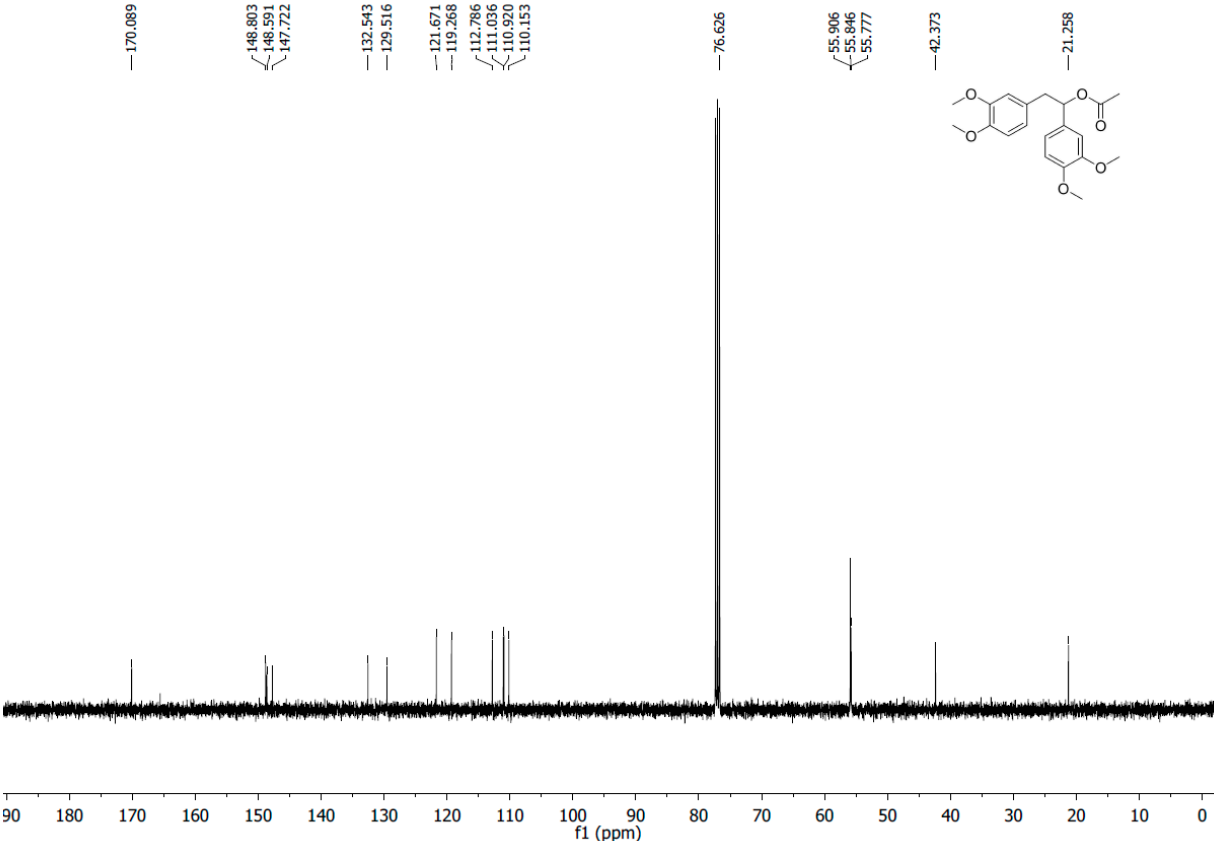

1,2-diphenylethyl benzoate (7)

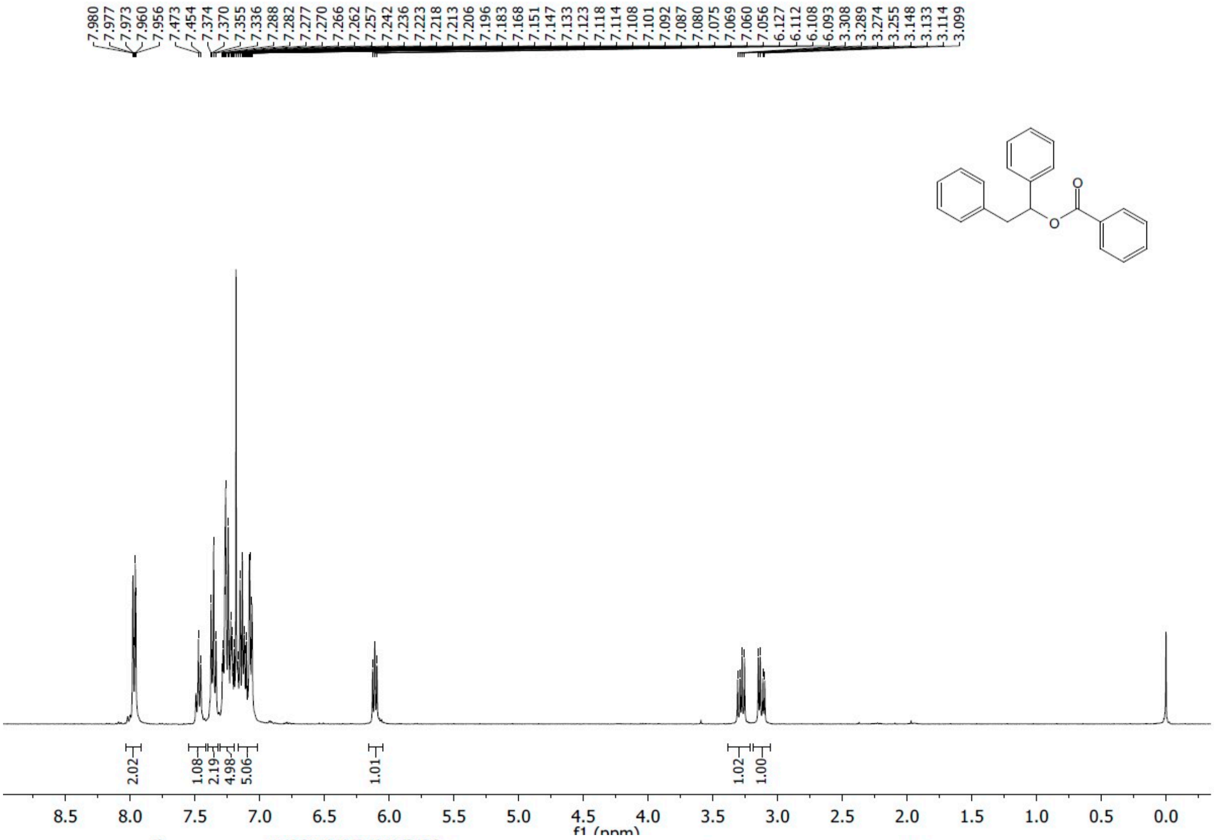

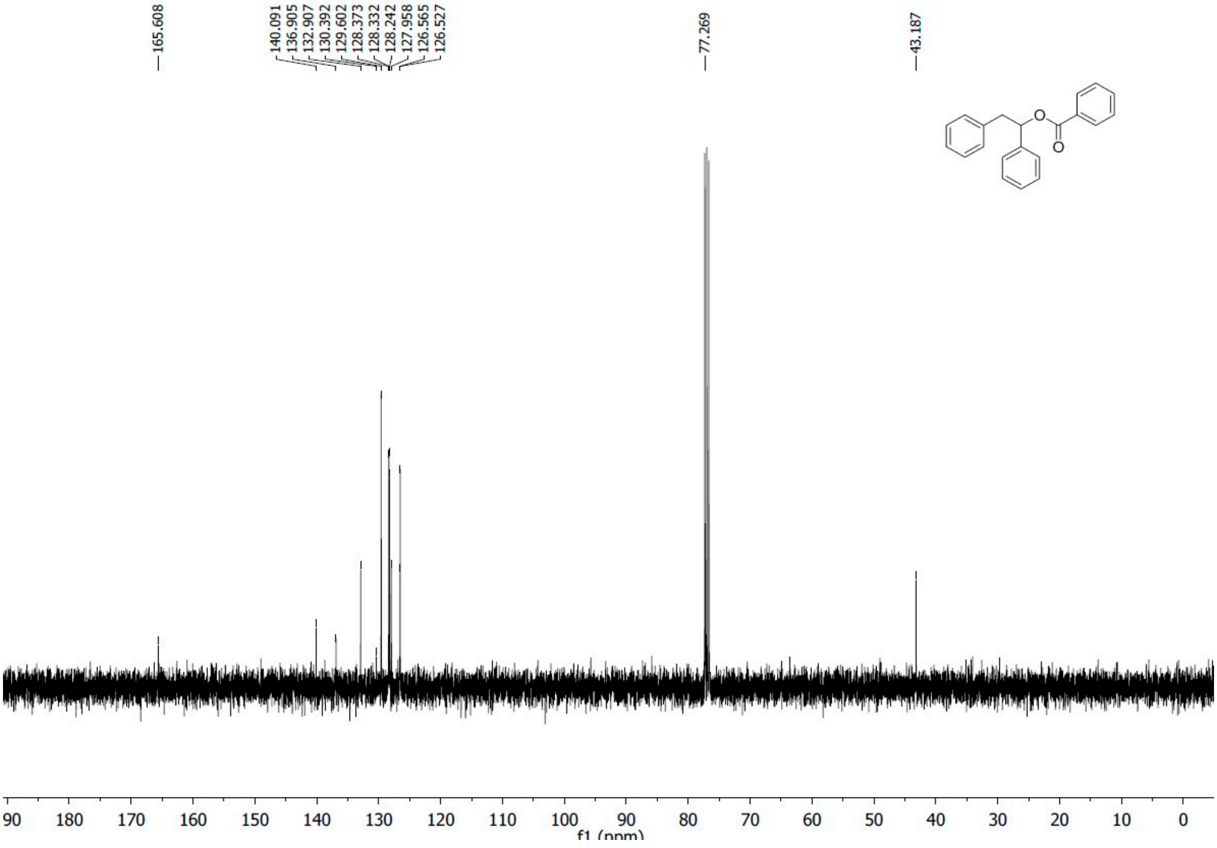

Supplement: Supplementary file 1 [file molecules-29-02268-s001.zip › molecules-2993914-supplementary.pdf]
